# A possible involvement of Nrf2-mediated heme oxygenase-1 up-regulation in protective effect of the proton pump inhibitor pantoprazole against indomethacin-induced gastric damage in rats

**DOI:** 10.1186/1471-230X-12-143

**Published:** 2012-10-16

**Authors:** Ho-Jae Lee, Young-Min Han, Eun-Hee Kim, Yoon-Jae Kim, Ki-Baik Hahm

**Affiliations:** 1 Lab of Chemoprevention, Lee Gil Ya Cancer and Diabetes Institute, Gachon University, Incheon, 406-840, Korea; 2 Lab of Translational Medicine, College of Pharmacy, CHA University, 605 Yeoksam 1-dong, Gangnam-gu, Seoul, 135-081, Korea; 3 Department of gastroenterology, Gachon University Gil Medical Center, Incheon, 405-760, Korea; 4 Lab of Translational Medicine, Department of Gastroenterology, CHA University Bundang Hospital, Seongnam, 463-838, Korea

**Keywords:** Heme oxygenase-1, Proton pump inhibitor, NSAIDs-induced gastropathy, Nrf2

## Abstract

**Background:**

Proton pump is an integral membrane protein that is ubiquitous ATP binding cassette (ABC) involved in many transport processes in all living organisms, among which a specialized form of pump, so called *p*-type proton pump, exists in the parietal cells of stomach. Though proton pump inhibitors (PPIs) are frequently prescribed to prevent nonsteroidal anti-inflammatory drugs (NSAIDs)-induced gastric damage, the acid suppressive actions do not suffice to explain.

**Methods:**

In order to document the effects of pantoprazole, one of PPIs, on the NSAIDs-induced gastric damage, *in vitro* and *in vivo* studies were performed. Immunocytochemistry, Western blot analysis, electrophoretic mobility shift assay and RT-PCR were conducted to evaluate the induction of heme oxygenase-1 (HO-1) through Nrf2 activation in normal gastric mucosal RGM-1 cells or *in vivo* stomach tissues from rats treated with indomethacin and/or pantoprazole.

**Results:**

Pantoprazole activated Nrf2 through inactivation of Keap1, after which the expression of HO-1 was significantly increased in a dose-dependent manner in RGM-1 cells. Increased ARE-DNA binding activity was observed maximally at 1 h with 300 μM of pantoprazole. The expression of HO-1 induced by pantoprazole was significantly associated with the increased *in vitro* tube formation (*P* < 0.05) and angiogenic factors including VEGF, bFGF, and HIF-1α. Indomethacin markedly increased the expressions of TNF-α, IL-1ß, IL-8, NOX-1, ICAM-1 and VCAM, whereas pantoprazole significantly decreased the expressions of indomethacin-induced these inflammatory mediators in accord with pantoprazole-induced HO-1 (*P* < 0.05) as documented with HO-1 inhibitor. *In vivo* model of indomethacin-induced gastric damage could validate *in vitro*-drawn results that pantoprazole remarkably protected against indomethacin-induced gastric damage, in which zinc protoporphyrin (5 mg/kg, *ip*) significantly abolished the protective efficacy of pantoprazole.

**Conclusion:**

These results demonstrate that Nrf2-mediated HO-1 induction of PPIs afforded a significant protective effect against NSAIDs-induced gastric damage beyond acid suppressive actions.

## Background

NSAIDs have huge prescription volumes mostly based on the following two benefits, one is an increase of aged patients necessitating NSAIDs prescription to relieve degenerative change-induced pain and the other is an additional trial for either the prevention of colon polyps or the escape from ischemic cardiovascular diseases [1,2]. However, the vast use of NSAIDs is limited by troublesome adverse effects such as the gastric erosion/ulcer, complicated bleeding from ulcers, and more serious complications arising at the small intestine and colon. Since the pharmacological action of NSAIDs is through the inhibition of prostaglandin (PG) synthesis via the suppression of cyclooxygenases (COX) [3], indiscernible diminution of gastroprotective prostaglandin E_2_ (PGE_2_) is responsible for these gastrointestinal (GI) adverse effects. Though the invention of selective COX-2 inhibitor, coxib, to guarantee GI safety has been suggested as the solving strategy, this solution also needs to improve. Although NSAIDs are used as potent anti-ulcer drugs, the additional uncovered mechanisms of NSAID toxicity [4,5] lead us to develop more potent and safer agents.

In the present time, the best choice for preventing NSAIDs-related GI toxicity is either the combination of NSAIDs and proton pump inhibitors (PPIs) or the choice of coxibs [6,7]. Since the healing rate of GI ulcers during continuous use of NSAIDs was greater in PPIs group than histamine type 2 receptor antagonist (H2-RA) group, PPIs have been preferred than H2-RA to cope with the adverse effect of NSAIDs [8,9]. Besides fundamental acid suppressive actions of PPIs, several functions have been revealed which are the reduction of pro-apoptotic signaling, acid-independent restoration of proliferating and repairing pathways [10], a reduction in mucosal oxidative damage, healing promoting action, and endoplasmic reticulum stress relieving mechanism [11-16]. Hahm *et al.*[17-19] have also reported that PPIs show the potential activities as anticancer therapeutics based on selective induction of apoptosis, anti-angiogenesis against *Helicobacter pylori*-associated carcinogenesis, and direct anti-mutagenic actions during tumorigenesis. However the mechanisms responsible for the protective effects of PPIs in NSAIDs-induced gastric damage remain to be determined.

Heme oxygenase-1 (HO-1), an inducible for the first and rate-limiting enzyme of heme degradation, has been known to protect against the cytotoxicity of oxidative stress and apoptotic cell death as well as inflammatory condition [20,21]. Fundamental protective effects of HO-1 against inflammation are mediated via anti-oxidative heme degradation, but also associated with the production of the anti-inflammatory mediators, for which redox dependent transcriptional activator, NF-E2-related factor 2 (Nrf2), and its phosphorylation/activation, and oxidation of Kelch-like ECH-associating protein 1 (Keap1) is mechanistically suggested [22-24]. Since the expression of HO-1 has been induced by anti-oxidative, anti-inflammatory, and ischemic relieving responses, in the current study, we hypothesized that the protective effects of PPIs against NSAIDs-induced gastric damage may be related to HO-1 and consequent angiogenesis beyond innate acid suppression. Altogether, our results demonstrate the novel mechanisms that PPIs induce the expression of HO-1 through activating Nrf2/inactivating Keap1 accompanied with the remuneration of ischemic change and the attenuation of inflammatory mediators, thereby facilitating protection against indomethacin-induced gastric damage.

## Methods

### Materials and cell cultures

Indomethacin was purchased from Sigma Aldrich (Saint Louis, MO) and pantoprazole was provided from Amore Pacific Pharmaceutical Co. (Seoul, Korea). Antibodies for β-actin, HO-1, α-tubulin, Keap1, Nrf2 and VEGF were all obtained from Santa Cruz Biotechnology (Santa Cruz, CA). Normal rat gastric mucosal RGM-1 cells were provided by Prof. Hirofumi Matsui, MD, PhD (Tsukuba Univ., Japan), were cultured in Dulbecco’s modified Eagle’s medium (DMEM) containing 10% (*v*/*v*) fetal bovine serum, 100 U/ml penicillin. Human umbilical vascular endothelial cells (HUVECs) were cultured in M199 medium (InnoPharma Screen, Seoul, Korea). Cells were maintained at 37°C in a humidified atmosphere containing 5% CO_2_. Appropriate amounts of RGM-1 cells or HUVECs were seeded and incubated for 24 h, then they were treated with the indicated dose of pantoprazole or indomethacin and incubated for the indicated times. HUVECs were moved to 1% O_2_ and 5% CO_2_ hypoxia chamber and incubated for 0–12 h for the *in vitro* tube formation assay.

### Electron spin resonance (ESR) spectroscopy and ROS generation measurement

Various concentrations of pantoprazole added to a total volume of 200 μl containing 0.05 mM FeSO_4_, 1 mM H_2_O_2_, 1 mM 5,5-dimethylpyrroline-N-oxide (DMPO, Sigma Aldrich, Saint Louis, MO), and 50 mM sodium phosphate at pH 7.4 at room temperature. Reactions were initiated by adding H_2_O_2_. After incubation for 1 min, aliquots of the reactions were transferred to a quartz cell and the spectrum of DMPO-OH was examined using an ESR spectrophotometer (JES-TE300, JEOL, Tokyo, Japan) under the following conditions: magnetic field, 338.0 ± 5.0 mT; microwave power, 4.95 mW; frequency, 9.421700 GHz; modulation amplitude, 5 mT; sweep time, 0.5 min; and time constant, 0.03 s. Cellular ROS contents were measured by incubating the control or pantoprazole treated RGM-1 cells with 10 μM H_2_DCF-DA (Invitrogen Life Technologies, Carlsbad, CA) for 30 min. Fluorescence was measured using a confocal laser microscope (LSM710, Carl Zeiss, Oberkochen, Germany).

### Western blot analysis

Treated cells were washed twice with PBS and then lysed in ice-cold cell lysis buffer (Cell Signaling Technology) containing 1 mM phenylmethylsulfonyl fluoride (PMSF, Sigma Aldrich). Proteins in lysates were separated by SDS-PAGE and transferred to polyvinylidene fluoride (PVDF) membranes, which were incubated with primary antibodies, washed, incubated with peroxidase-conjugated secondary antibodies, rewashed, and then visualized using an enhanced chemiluminescence (ECL) system (GE Healthcare, Buckinghamshire, UK).

### Electrophoretic mobility gel shift assay (EMSA)

Nuclear and cytoplasmic fractions were extracted using NE-PER Nuclear and cytoplasmic reagents (Pierce, Rockford, IL), according to the manufacturer’s instructions. Antioxidant response element (ARE) oligonucleotide probe, 5′-TTT TCT GCT GAG TCA AGG TCC G-3′**,** and HIF-1α oligonucleotide probe, 5′-TCT GTA CGT GAC CAC ACT CAC CTC-3′, was labeled with [γ-^32^P] ATP using T4 polynucleotide kinase (Promega, Madison, WI) and separated from unincorporated [γ-^32^P] ATP by gel filtration using a nick spin column (GE Healthcare). Before adding the ^32^P-oligonucleotide (1x10^5^ cpm), 10 μg of nuclear extract was kept on ice for 15 min in gel shift binding buffer. To determine the sequence specificity of the NF-κB DNA interaction, we added an excess of unlabeled oligonucleotides. After 20 min of incubation at room temperature, 2 μl of 0.1% bromophenol blue was added, and samples were electrophoresed through 6% non-denaturing PAGE at 150 V in a cold room. Finally, gels were dried and exposed to X-ray film (Kodak, Rochester, NY).

### Immunocytochemistry

Treated cells in chamber slides were fixed by 3.7% formaldehyde for 15 min. After washing, cells were blocked in 5% BSA solution containing 0.1% Triton X-100 in PBS for 1 h at room temperature, and then incubated with primary antibody (1:100) for 12 h at 4°C. Cells were then washed 3 times, incubated with secondary antibody (1:300) for 1 h, and then with 4′-6-diamidino-2-phenylindole (DAPI, 100 ng/ml) for 1 min at room temperature. After washing 3 times, cells were mounted with Prolong Gold antifade reagent (Invitrogen Life Technologies, Carlsbad, CA). Fluorescence was visualized under a confocal laser microscope (LSM710, Carl Zeiss).

### RNA isolation and quantitative reverse transcription polymerase chain reaction (qRT-PCR)

After treatment, media was removed by suction and cells were washed with Dulbecco’s phosphate-buffered saline (DPBS) twice. RiboEX (Gene All, Seoul, Korea) was added to plates, which were then incubated for 10 min at 4°C. RiboEX was harvested and placed in a 1.5 ml tube, and chloroform was added and gently mixed. After incubation for 10 min in ice, samples were centrifuged at 10,000 × g for 30 min. Supernatants were extracted and mixed with isopropanol, and mixtures were incubated at 4°C for 1 h. After centrifuging at 13,000 *g* for 30 min, pellet was washed with 70% (*v*/*v*) ethanol. After allowing the ethanol to evaporate completely, pellets were dissolved in diethylene pyrocarbonate (DEPC)-treated water (Invitrogen Life Technologies, Carlsbad, CA). cDNA was prepared using reverse transcriptase derived from murine Maloney leukemia virus (Promega, Madison, WI), according to the manufacturer’s instructions. PCR was performed over 30 cycles of: 94°C for 20 sec, 58°C for 30 sec, and 72°C for 45 sec. Oligonucleotide primers for PCR (Table 1) were purchased from Bioneer (Daejeon, Korea). All qRT-PCR experiments were repeated in triplicate and quantification was shown in mean ± SD.

**Table 1:** The sequence of PCR primers

	
GAPDH	Forward 5′-GGT GCT GAG TAT GTC GTG GA -3′
	Reverse 5′-TTC AGC TCT GGG ATG ACC TT-3′
HO-1	Forward 5′-GAG AGC ATG TCC CAG GAT TT-3′
	Reverse 5′-GGT TCT GCT TGT TTC GCT CT -3′
COX-2	Forward 5′-GAA ATG GCT GCA GAG TTG AA -3′
	Reverse 5′-TCA TCT AGT CTG GAG TGG GA -3′
HIF-1α	Forward 5′-AAC AAA CAG AAT CTG TCC TC-3′
	Reverse 5′-GGT AAT GGA GAC ATT GCC AG-3′
VEGF	Forward 5′-CAA TGA TGA AGC CCT GGA GT-3′
	Reverse 5′-GAT TTC TTG CGC TTT CGT TT -3′
PDGF	Forward 5′-AGG AAG CCA TTC CCG CAG TT-3′
	Reverse 5′-CTA ACC TCA CCT GGA CCT CT -3′
bFGF	Forward 5′-TAT GAA GGA AGA TGG ACG GC-3′
	Reverse 5′-AAC AGT ATG GCC TTC TGT CC -3′
IL-1β	Forward 5′-CAT TGT GGC TGT GGA GAA G-3′
	Reverse 5′-ATC ATC CCA CGA GTC ACA GA -3′
IL-8	Forward 5′-CAG ACA GTG GCA GGG ATT CA-3′
	Reverse 5′-TTG GGG ACA CCC TTT AGC AT-3′
TNF-α	Forward 5′-TAC TGA ACT TCG GGG TGA TT -3′
	Reverse 5′-CAG CCT TCT CCC TTG AAG AG-3′
ICAM-1	Forward 5′-TGT GCT TTG AGA ACT GTG GC-3′
	Reverse 5′-GGT TCT GTC CAA CTT CTC AG -3′
VCAM-1	Forward 5′-GAG ACA AAA CAG AAG TGG AAT-3′
	Reverse 5′-TAC AAG TGG TCC ACT TAT TTC -3′
NOX1	Forward 5′-GAG AAA TTC TCG GAA CTG CC-3′
	Reverse 5′-TGT TGG CTT CTA CTG TAG CG -3′

### *In vitro* angiogenesis assay

This assay was performed using a commercial kit according to the manufacturer’s instruction (Millipore, Billerica, MA). For hypoxic conditions, ECMatrix and Diluent buffer were mixed to make a solid gel, which was then plated in 96 well microplates. Human umbilical vein endothelial cells (HUVEC, 1.0 × 10^5^/ml) were seeded with control, Indomethacin, indomethacin plus pantoprazole, indomethacin plus pantoprazole plus ZnPPIX incubated at 37°C for 4 h. Tube formation was observed under a light microscope.

### Indomethacin-induced gastric damage model

A total of 48 rats were purchased from Charles River (Osaka, Japan). Animals were handled in an accredited animal facility in accordance with Association for Assessment and Accreditation of Laboratory Animal Care International (AAALAC International) guidelines under the facility named CACU (The Center of Animal Care and Use) of Lee Gil Ya Cancer and Diabetes Institute, Gachon University after Institutional Ethics Review Board approval. Animals were divided into four groups each consisting of 12 rats and were starved for 24 h before the experimentation as follows; all rats were administered with intraperitoneal injection of indomethacin (10 mg/kg) to provoke gastric damages, second group with intraperitoneal injection of 10 mg/kg of pantoprazole, the third group with intraperitoneal injection of 30 mg/kg of pantoprazole, and the fourth group with intraperitoneal injection of 30 mg/kg of pantoprazole and 5 mg/kg of ZnPPIX to inhibit the activity of HO-1. Animals were sacrificed 16 h after each administration. The stomachs of rats were removed and opened along the greater curvature and then washed with ice cold phosphate buffered solutions. The numbers of either erosions or ulcers were determined under the magnified photographs. Homogenates obtained from scratched gastric mucosa were kept into liquid nitrogen tank until the assay.

### Statistical analysis

Results are expressed as the mean ± SD. The data were analyzed by one-way ANOVA, and the statistical significance between groups was determined by Duncan’s multiple range test. Statistical significance was accepted at *P* < 0.05.

## Results

### Pantoprazole increased HO-1 expression through Nrf2 activation

**Figure 1:**
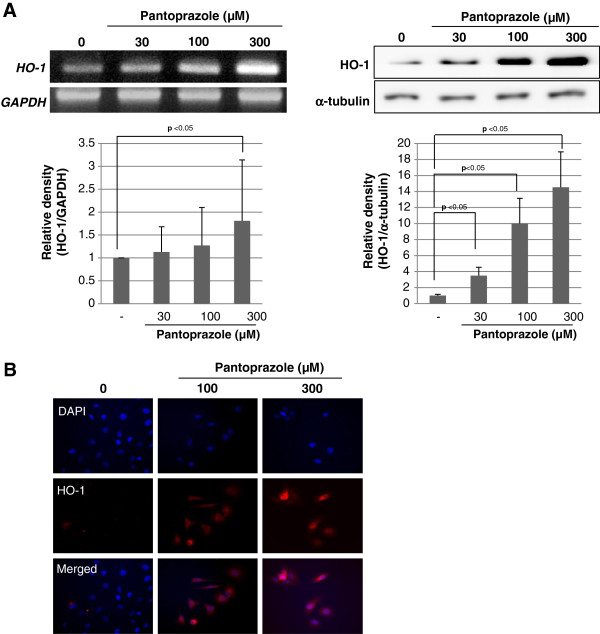
**Pantoprazole induced the expression of HO-1. (A)** RT-PCR and Western blot for HO-1 expression according to different dosing of pantoprazole, 30, 100, and 300 μM, respectively. These figures are representatives of the results obtained in 3 independent experiments. **(B)** Confocal imaging of HO-1. Pantoprazole increased the expression of HO-1 in a dose-dependent manner in normal rat gastric mucosal RGM-1 cells.

**Figure 2:**
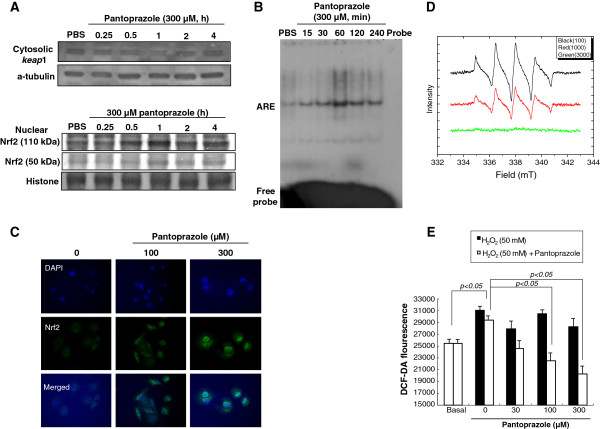
**Pantoprazole increased Nrf2 nuclear translocation and cytoplasmic keap1 inactivation, led to significant anti-oxidation. (A)** Western blots for either cytosolic Keap1 or nuclear Nrf2 in a different time in the presence of 300 μM pantoprazole. **(B)** Electrophoretic mobility shift assay (EMSA) for ARE-DNA binding activity. Significantly increased DNA-binding of Nrf2 was noted 60 min after pantoprazole administration. **(C)** Confocal imaging of Nrf2 after different dosing of pantoprazole, 100 μM and 300 μM. **(D)** Electron spin resonance (ESR) measurement for Fenton reaction-generated hydroxyl radicals. **(E)** The changes of DCF-DA fluorescence after different dosing of pantoprazole, 30, 100, and 300 μM. Pantoprazole decreased significantly H_2_O_2_-induced oxidative fluorescence.

Based on preliminary study, we have found pantoprazole showed the highest induction of HO-1 expression in RGM-1 cells among PPIs, lansoprazole, rabeprazole, omeprazole, and pantoprazole (data not shown). Pantoprazole increased the expression of HO-1 mRNA and protein in a dose-dependent manner (Figure 1A) and immunocytochemical analysis also revealed that the level of HO-1 was significantly increased by pantoprazole treatment in the cytoplasm of RGM-1 cells in a dose-dependent manner (Figure 1B). To understand the mechanisms underlying the up-regulation of HO-1 expression by pantoprazole, we examined its effects on the activation of Nrf2, a major transcription factor that mediates the ARE/EpRE-driven expression of antioxidant enzymes. When we measured the expression level of keap1, a repressor of Nrf2 in cytoplasmic fraction treated with 300 μM pantoprazole in a different time point, significantly decreased expression of Keap1 was observed in 1 h (Figure 2A), suggesting that HO-1 might be transcribed after pantoprazole treatment through Nrf2 activation and Keap1 inactivation. As illustrated in Figure 2A, Nrf2 nuclear co-localization was evident in RGM-1 cells treated with pantoprazole. The maximum ARE-binding activity of Nrf2 was increased in 1 h as well as its nuclear localization induced by pantoprazole (Figure 2B). To ascertain the nuclear accumulation of Nrf2, we conducted an immunocytochemical analysis using the anti-Nrf2 antibody. As shown in Figure 2C, Nrf2 was translocated into nucleus with 300 μM pantoprazole treatment. These results consistently suggested that pantoprazole could increase HO-1 levels through transcriptional activation of Nrf2 in rat gastric epithelial cells.

Since the biological significance of HO-1 pantoprazole possesses an anti-oxidative action related to HO-1 induction. ESR measurement was ultimate way to trace free radical generation using spin adduct, for which we generated hydroxyl radicals using DMPO as an adductor under Fenton reaction. As seen in Figure 2D, 3 mM pantoprazole exerted clear scavenging action of DMPO-adduct-generating hydroxyl radicals. Since ESR measurement was executed in chemical reaction condition not biological system and pantoprazole is not a professional antioxidant, it definitely contributed to scavenge hydroxyl radicals even though it is relatively high concentration of pantoprazole to scavenge reactive oxygen species. These chemical results from ESR study were further validated with biological test using DCF-DA fluorescence measurement, showing that pantoprazole showed significant DCF-DA reduction in a dose-dependent manner (*P* < 0.05, Figure 2E).

### Angiogenic actions consequent to pantoprazole-induced HO-1

**Figure 3:**
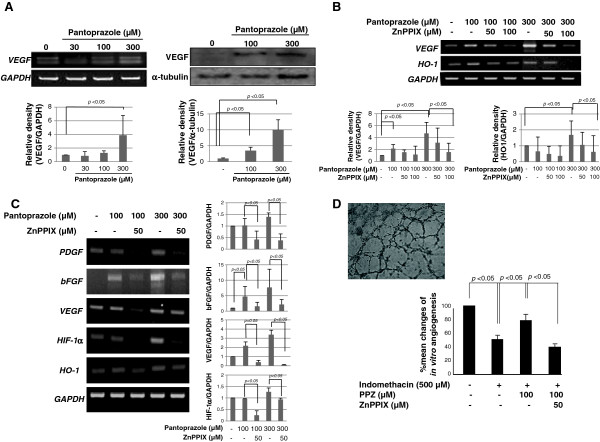
**Pantoprazole induced angiogenic factors related to HO-1 induction. (A)** RT-PCR and western blot for VEGF. Significant inductions of VEGF were noted with pantoprazole, significantly after 300 μM pantoprazole (*p<0.05*). These figures are representatives of the results obtained in 3 independent experiments. **(B)** The changes of VEGF and HO-1 with pantoprazole alone or combination of pantoprazole and ZnPPIX. The induction of VEGF after pantoprazole was significantly attenuated with HO-1 inhibitor, signifying the implication of HO-1 in PPI-induced VEGF. These figures are representatives of the results obtained in 3 independent experiments. **(C)** Other angiogenic factors related to pantoprazole. bFGF and VEGF was increasingly expressed with pantoprazole, but these inductions were attenuated with HO-1 inhibitor. These figures are representatives of the results obtained in 3 independent experiments. **(D)***in vitro* angiogenesis assay. Tube formation of HUVEC was significantly decreased with 50 μM indomethacin. Pantoprazole compensated indomethacin-induced defective angiogenesis as assessed with the percentage of tube formation, whereas HO-1 inhibitor cancelled these overcome of pantoprazole-induced angiogenesis. These figures are representatives of the results obtained in 3 independent experiments.

PPIs increased the expressions of VEGF mRNA and protein in RGM-1 cells (Figure 3A). In order to document whether the incremental induction of VEGF with PPIs is related to HO-1 induction, we repeated the experiments with PPI alone or co-treatment of pantoprazole and zinc protoporphyrin (ZnPPIX) as HO-1 inhibitor. As results, we could reconfirm the expression of VEGF induced by pantoprazole and incremental co-treatment of ZnPPIX clearly decreased the expression of PPI-induced VEGF in a dose-dependent manner (Figure 3B). Next, we investigated the changes of representative angiogenic factors, bFGF, PDGF, and HIF-1α and observed these expressions relevant to pantoprazole-induced HO-1 in Figure 3C. As anticipated, pantoprazole significantly increased these expressions of several angiogenic factors. Also these inductions of bFGF and PDGF were significantly attenuated with co-treatment of pantoprazole and ZnPPIX. In order to verify whether these inductions of angiogenic factor after pantoprazole were actually related to angiogenesis, *in vitro* angiogenesis assay was done (Figure 3D). Tube formation was significantly attenuated with 500 μM indomethacin in HUVECs, whereas 100 μM pantoprazole could overcome these derangements of angiogenesis treated with indomethacin. However, additional treatment with ZnPPIX abolished these benefits of overcoming angiogenesis by pantoprazole under indomethacin administration. These results suggest that pantoprazole compensated NSAIDs-induced defective angiogenesis through its HO-1 induction capability.

### Pantoprazole-induced HO-1 attenuated indomethacin-associated inflammatory assault

**Figure 4:**
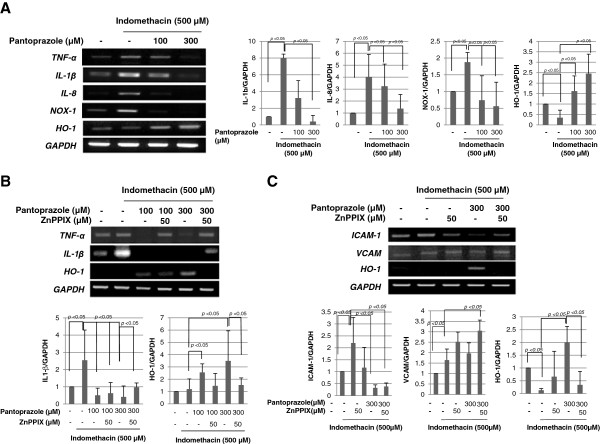
**Pantoprazole attenuated indomethacin-induced inflammatory mediators through HO-1. (A)** RT-PCR for TNF-α, IL-1β, IL-8, and HO-1. 500 μM indomethacin increased the expression of inflammatory mediators including TNF-α, IL-1β, IL-8, but significantly decreased HO-1 expression. NOX-1 was significantly increased with indomethacin. These figures are representatives of the results obtained in 3 independent experiments. **(B)** Pantoprazole significantly decreased indomethacin-induced IL-1β and TNF-α, but these beneficiary actions of pantoprazole were abolished with ZnPPIX, leading to the conclusion that anti-inflammatory actions of pantoprazole were owing to HO-1 induction. These figures are representatives of the results obtained in 3 independent experiments. **(C)** Pantoprazole significantly decreased indomethacin-induced ICAM-1 and VCAM in HUVEC cells, but these beneficiary actions of pantoprazole were also abolished with ZnPPIX as HO-1 inhibitor. These figures are representatives of the results obtained in 3 independent experiments.

Though indomethacin is an anti-inflammatory agent, it has been known that NSAIDs induced gastric damage through increased expressions of inflammatory mediators including TNF-α, IL-1β, IL-8, and NADPH oxidase-1 (NOX-1). As seen in Figure 4A, 500 μM indomethacin significantly increased the expressions of TNF-α, IL-1β, and IL-8 in gastric epithelial cells, whereas it decreased the expression of HO-1, confirming that inflammatory mediators might be mechanistically associated with indomethacin-induced gastric epithelial cell damage. In this condition, co-administration of 500 μM indomethacin and 300 μM pantoprazole significantly decreased the expressions of TNF-α, IL-1β, and IL-8 accompanied with statistically significant increases of HO-1 expression. Additionally, indomethacin challenge significantly increased the expressions of NOX-1, in which pantoprazole significantly attenuated the increased expression of NOX-1, suggesting that pantoprazole-induced HO-1 imposed anti-inflammatory actions under indomethacin. Next, in order to further prove whether these attenuations of inflammatory mediators after pantoprazole are resulted from HO-1 induction, we repeated these experiments with ZnPPIX administration. As seen in Figure 4B, the co-treatment of ZnPPIX in the presence of pantoprazole significantly abolished the benefits of anti-inflammatory action. The incremental inductions of ICAM-1 and VCAM after indomethacin treatment were responsible for ischemia and aggravated organ damages, in which either vascular inflammation or increased leukocyte aggregation was engaged. Treatment with indomethacin significantly increased the expressions of ICAM-1 and VCAM in HUVEC, but co-challenge of indomethacin and pantoprazole significantly decreased indomethacin-induced ICAM-1 expression relevant to increased induction of HO-1 (Figure 4C). In this condition, ZnPPIX did not impose the attenuation of ICAM or VCAM in spite of pantoprazole treatment, further assuring the beneficiary action of pantoprazole associated with HO-1 induction.

### Pantoprazole-induced HO-1 enfeebled indomethacin-induced gastric injury

**Figure 5:**
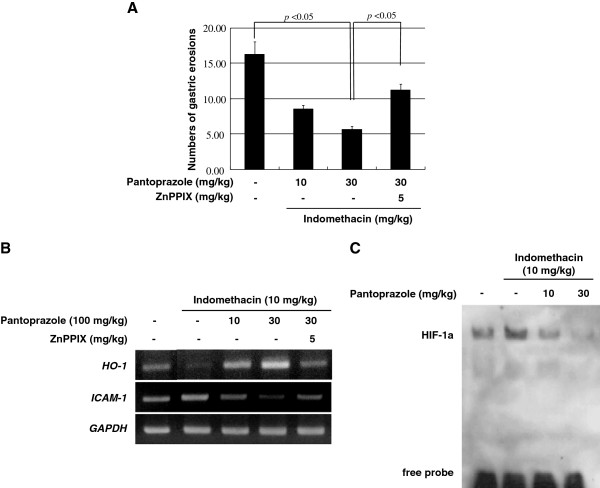
**Pantoprazole prevents to the indomethacin-induced gastric damage through depending on the induction of HO-1. (A)** Mean index of indomethacin-induced gastric damages. **(B)** RT-PCR for HO-1 and ICAM-1 according to group. **(C)** EMSA for HIF-1α according to group. Indomethacin significantly increased HIF-1α-DNA binding, whereas pantoprazole significantly decreased HIF-1α-DNA binding in a dose-dependent manner.

To investigate the protective effect of pantoprazole *in vivo*, indomethacin (10 mg/kg) was administered intraperitoneally for 16 h in rats. Treatment with indomethacin significantly provoked gastric mucosal injuries including hemorrhagic erosions and ulcerations. However, these gastric pathologies provoked with indomethacin were significantly attenuated with intraperitoneal injection of 30 mg/kg pantoprazole (Figure 5A). Since these protective effects of 30 mg/kg pantoprazole against indomethacin-induced gastric damages were significantly abolished with co-treatment of intraperitoneal administered 5 mg/kg ZnPPIX, we confirmed that the protective action of pantoprazole was based on its capability of HO-1 induction. When the expressions of HO-1 and ICAM-1 were measured in mucosal homogenates from each group, indomethacin administration significantly decreased the expressions of HO-1 and increased the expression of ICAM-1. However, owing to increased HO-1 expressions after pantoprazole as well as significantly attenuated levels of ICAM-1, indomethacin-induced gastric damage was proportionally improved in spite of indomethacin challenge (Figure 5B). To demonstrate how pantoprazole improved the defective angiogenesis and ischemia provoked by indomethacin, EMSA using HIF-1α was performed (Figure 5C). The HIF-1α-DNA binding activity was highly increased in indomethacin alone group, which was significantly decreased in gastric homogenates of pantoprazole treatment group, signifying pantoprazole significantly relieved hypoxic condition induced by indomethacin. Taken together, PPIs exerted strong protection against indomethacin-induced gastric mucosal damage through significant HO-1 induction, which is beyond authentic acid suppressive action, though we did not measure the changes of gastric acidity.

## Discussion

Before the current investigation, several researchers have published that PPIs could prevent gastric mucosal injury by other mechanisms beyond the action of its specific acid inhibition [11,14,25-28], we could add more evidences regarding protective action of PPIs against NSAIDs. Pantoprazole significantly induced the expression of HO-1 through Nrf2 activation relevant to anti-inflammatory, anti-oxidative, and ischemia relieving actions. Novel finding of this study different from other investigations is that pantoprazole shows protective actions to the NSAIDs-induced injury through either the correction of angiogenic handicap or the attenuation of inflammation propensity.

Becker JC *et al.*[16] demonstrated that both omeprazole and lansoprazole protected human gastric epithelial and endothelial cells against oxidative stress similar to our study, but used different kinds of PPIs. Since this effect was abrogated in the presence of the HO-1 inhibitor, ZnPPIX, HO-1 seems to be a right target of PPIs in both endothelial and gastric epithelial cells. In this study, we observed untouched novel finding that HO-1 induced by PPIs decreased NSAIDs-incurred inflammation and angiogenic derangement. Takagi T *et al.*[25] also investigated the role of Nrf2, its phosphorylation/activation, and oxidation of Keap1 in lansoprazole-induced HO-1 up-regulation using same cell line with us, RGM-1 cells. When RGM-1 cells were transfected with HO-1 enhancer luciferase reporter plasmid containing mutant stress response element, lansoprazole-induced HO-1 reporter gene activity was not increased. Taken together with our results, lansoprazole or pantoprazole up-regulated HO-1 expression and this up-regulated HO-1 contributed to the anti-inflammatory effects. Against gastric damage induced by NSAIDs, PPIs significantly reduced the mRNA expression and production of TNF-α and IL-1β in THP-1 cells stimulated by other irritants such as lipopolysaccharide (LPS) and *H. pylori* water extracts [15]. Lansoprazole inhibited the phosphorylation and degradation of inhibitory factor kappaB-alpha (IκBα) and phosphorylation of ERK in THP-1 cells, reaching to the conclusion that PPIs could exert anti-inflammatory effects by directly suppressing induction of TNF-α and IL-1β via the inhibition of NF-κB and ERK activation in inflammatory cells. Though they used inflammatory cells and we used gastric mucosal cells, we observed similar results that PPIs-induced HO-1 significantly inhibited indomethacin-induced levels of TNF-α and IL-1β. Natale G *et al.*[14] have reported that pretreatment with 90 μM/kg lansoprazole significantly prevented alcohol-induced gastric damage, suggesting a significant reduction of gastric oxidative stress associated with an increased bioavailability of mucosal sulfhydryl (SH) compounds.

Then, the question arises whether there might be difference in PPIs-induced protective mechanisms according to kinds of NSAIDs or kinds of PPIs. Blandizzi C *et al.*[29] treated male Sprague–Dawley rats with several kinds of NSAIDs, 100 μM/kg indomethacin, 60 μM/kg diclofenac, 150 μM/kg piroxicam or 150 μM/kg ketoprofen. Thirty minutes before NSAIDs, animals were orally treated with lansoprazole and four hours after the end of treatment, gastric mucosal PGE_2_, malondialdehyde (MDA), myeloperoxidase (MPO) or non-protein sulfhydryl compounds (GSH) levels were measured, respectively. As result, PPIs prevented against NSAIDs-induced gastric damage irrespective of kinds of NSAIDs, mainly alleviating NSAIDs-induced mucosal oxidative injury [28]. In preliminary study, we have also tested the HO-1 inducing capacity according to PPIs including lansoprazole, pantoprazole, rabeprazole, and omeprazole and we have found pantoprazole was the best in inducing HO-1 as well as other biological actions. We speculated these differences were based on the stability of PPI in aqueous condition.

Interestingly, these protective actions of PPIs against NSAIDs-induced gastric damages were not confined to just stomach. According to Takagi T *et al.*[27], they investigated whether PPIs ameliorated intestinal mucosal injuries induced by ischemia-reperfusion in rats and found that lansoprazole or pantoprazole have been demonstrated to prevent gastrointestinal mucosal injury by mechanisms independent of acid inhibition. Esomeprazole also counteracted the detrimental action of indomethacin on ulcer repair through both acid-dependent and acid-independent mechanisms [26]. Though subtle difference in the action mechanisms related to acid suppression, PPIs irrespective of kinds were similar in the protective action beyond acid suppression, but we have used pantoprazole in our investigation, which shows utmost protective actions against NSAIDs-induced gastric damages based on HO-1 induction.

## Conclusion

We confirmed the new finding, never touched before, how pantoprazole can protect stomach from NSAIDs-induced gastric damage beyond authentic acid suppressive action and elucidated the mechanism that pantoprazole improved NSAIDs-induced ischemia and attenuated NSAIDs-associated gastric inflammation through Nrf2-driven HO-1 induction. Conclusively, PPIs in the current form or newer formulation based on reinforced action of PPIs-induced HO-1 expression will cover NSAIDs-induced gastric damage. However, more investigations will be prerequisite whether this kind of protection with PPIs can also be ascribed to NSAIDs-induced enteropathy although there were conflicting reports, that is, one study showed PPIs can aggravate NSAIDs-induced enteropathy through dysbiosis [30], but other study showed prevention by lansoprazole of indomethacin-induced small intestinal ulceration in rats through induction of HO-1 and carbon monoxide [31].

## Abbreviations

PPIs: Proton pump inhibitors; NSAIDs: Nonsteroidal anti-inflammatory drugs; HO-1: Heme oxygenase-1; COX-2: Cyclooxygenase-2; H2-RA: Histamine type 2 receptor antagonist; Coxib: Selective COX-2 inhibitor; Nrf2: NF-E2-related factor 2; Keap1: Kelch-like ECH-associating protein 1; *H. pylori*: *Helicobacter pylori*; VEGF: Vascular endothelial growth factor; ESR: Electron spin resonance; DMPO: 5,5-dimethylpyrroline-N-oxide; ZnPPIX: Zinc protoporphyrin; ARE: Antioxidant response element; EMSA: Electrophoretic mobility shift assay; NF-κB: Nuclear factor-kappaB; IκBα: Inhibitory factor kappaB-alpha; HUVEC: Human umbilical vein endothelial cell; LPS: Lipopolysaccharide; DCFDA: 2′,-7′-dichlorodihydrofluorescein diacetate; TNF-α: Tumor necrosis factor-alpha; OH: Hydroxyl radical; NOX: NADPH oxidase; SH: Sulfhydryl; HIF-α: Hypoxia inducing factor-alpha; DEPC: Diethylene pyrocarbonate; DMPO: 5,5-dimethyl-1-pyrroline N-oxide; GSH: Reduced glutathione.

## Competing interests

The authors declare that they have no competing interests.

## Authors’ contributions

KBH first set the hypothesis, mentored, and designed the whole experiments and my co-authors, who contributed to the completion of current manuscript, YMH as a PhD student and HJL, a professor in the lab of chemoprevention contributed to most experimental works. EHK and YJK contributed to complete the current study and manuscript. All authors read and approved the final manuscript.

## Pre-publication history

The pre-publication history for this paper can be accessed here:

http://www.biomedcentral.com/1471-230X/12/143/prepub
